# Bacterial transcriptional response to labile exometabolites from photosynthetic picoeukaryote *Micromonas commoda*

**DOI:** 10.1038/s43705-023-00212-0

**Published:** 2023-01-23

**Authors:** Frank X. Ferrer-González, Maria Hamilton, Christa B. Smith, Jeremy E. Schreier, Malin Olofsson, Mary Ann Moran

**Affiliations:** 1 Department of Marine Sciences, University of Georgia, Athens, GA 30602 USA; 2 Department of Aquatic Sciences and Assessment, Swedish University of Agricultural Sciences, 750 07 Uppsala, Sweden

**Keywords:** Biogeochemistry, Microbiology

## Abstract

Dissolved primary production released into seawater by marine phytoplankton is a major source of carbon fueling heterotrophic bacterial production in the ocean. The composition of the organic compounds released by healthy phytoplankton is poorly known and difficult to assess with existing chemical methods. Here, expression of transporter and catabolic genes by three model marine bacteria (*Ruegeria pomeroyi* DSS-3, *Stenotrophomonas* sp. SKA14, and *Polaribacter dokdonensis* MED152) was used as a biological sensor of metabolites released from the picoeukaryote *Micromonas commoda* RCC299. Bacterial expression responses indicated that the three species together recognized 38 picoeukaryote metabolites. This was consistent with the *Micromonas* expression of genes for starch metabolism and synthesis of peptidoglycan-like intermediates. A comparison of the hypothesized *Micromonas* exometabolite pool with that of the diatom *Thalassiosira pseudonana* CCMP1335, analyzed previously with the same biological sensor method, indicated that both phytoplankton released organic acids, nucleosides, and amino acids, but differed in polysaccharide and organic nitrogen release. Future ocean conditions are expected to favor picoeukaryotic phytoplankton over larger-celled microphytoplankton. Results from this study suggest that such a shift could alter the substrate pool available to heterotrophic bacterioplankton.

## Introduction

Phytoplankton are a main source of the bioavailable dissolved organic carbon (DOC) that supports heterotrophic marine bacteria. DOC can be released through phytoplankton exudation to maintain physiological balance, or through senescence, grazing, or viral lysis [1, 2]. Heterotrophic bacteria recycle this pool of labile dissolved compounds on time frames of hours to days [3–5], maintaining standing concentrations of individual metabolites in the nanomolar range or lower [5–9]. This presents substantial analytical challenges for studying phytoplankton metabolite utilization by heterotrophic bacteria. Untargeted chemical analyses have uncovered hundreds of thousands of organic features in surface ocean DOC [10], but very few have been identified as bacterioplankton substrates [11–13].

One important marine phytoplankton group contributing metabolites to the labile DOC pool of the surface ocean is the picophytoplankton. These small autotrophs have cell diameters ranging between 0.6 and 3 μm [14], and currently account for 10% to 40% of marine planktonic primary production [15, 16]. The most abundant of the eukaryotic picophytoplankton is the class Prasinophyceae (Chlorophyta) [14], and the three dominant genera in this group, *Micromonas*, *Ostreococcus*, and *Bathycoccus*, together represent more than 90% of picoeukaryote 18S rRNA sequences in ocean surveys [17]. *Micromonas commoda*, the focus of this study, is a cosmopolitan member of the Prasinophyceae [18] and is abundant in temperate coastal ecosystems [19–21]. Previous studies using chemical approaches have characterized the major carotenoids and lipids in this picophytoplankter [22] and the endometabolites of close relative *Micromonas pusilla* [23].

Our interest in characterizing *M. commoda* metabolites assimilated by heterotrophic bacteria stems from forecasted shifts in picoplankton community composition as the planet warms [24–27]. In fact, picophytoplankton are predicted to be favored in warmed seawater [24, 25, 27–29] due to the inverse relationship observed between temperature and phytoplankton cell size [25] and expectations that smaller cells are favored under low nutrient availability [16]. Experiments addressing effects of climate-relevant factors on picophytoplankton have shown significant positive growth responses to both increased temperature and seawater acidification [21, 30].

In this study we use model bacterial species as biological sensors of labile compounds released by the eukaryotic picophytoplankter *M. commoda* RCC299. Our approach identified cellular machinery for organic carbon acquisition that was transcriptionally activated by the bacteria when *M. commoda* served as the source of growth substrates. We focused mainly on changes in expression of bacterial transporters and catabolic genes, using gene annotations to generate hypotheses about the identity of picophytoplankton-derived metabolites. The experiments involved pairwise co-cultures in which *M. commoda* was the sole source of organic matter for each of three bacterial species selected to represent taxa commonly found associated with natural marine phytoplankton: *Ruegeria pomeroyi* DSS-3 (Rhodobacteraceae; isolated from Southeastern US coastal seawater), *Stenotrophomonas* sp. SKA14 (Gammaproteobacteria; isolated from Swedish coastal seawater), and *Polaribacter dokdonensis* MED152 (Flavobacteriales; isolated from the Mediterranean Sea). These same species were used in a previous study of *Thalassiosira pseudonana* CCMP1335 exometabolites (35), allowing a comparison of hypothesized organic matter release by the two phytoplankton.

## Methods

### Co-cultures

Axenic cultures of *Micromonas commoda* RCC299 (National Center for Marine Algae, NMCA) were grown in 1 L of organic-carbon free defined medium L1-Si [31] as modified by NCMA (https://ncma.bigelow.org/) at a salinity of 35 in 1900 mL vented polystyrene tissue culture flasks. Flasks were maintained at 18 ^o^C under 16 h light at 160 μmol photons m^−2^s^−1^ and 8 h dark. Pre-cultures of *Micromonas* were sequentially upscaled (50 ml, 200 ml, 1 L) with transfers occurring during the exponential growth phase. After growing for 7 d (early stationary growth phase; ~2.7 × 10^6^ cells ml^−1^), bacteria pre-grown in YTSS medium [32] were washed 5 times in sterile L1 medium at 6000 RCF and inoculated into the axenic cultures at ~10^6^ cells ml^−1^. Replicate co-cultures were established for each bacterial strain and also for an axenic phytoplankton control. Bacterial contamination of the axenic cultures was ruled out based on lack of colony formation from culture aliquots spread onto YTSS plates and absence of bacterial-size particles in flow cytometry scattergrams (see below). After incubation in the light for 8 h to allow for transcriptional changes in response to available substrates, cells were collected on 0.2 μm pore-size 47 mm Supor filters (500 ml per filter). Filters were immediately flash frozen in liquid N_2_ and stored at −80 ^o^C until processing. Three additional treatments were established with bacterial strains introduced individually into L1 medium with 400 μM C glucose as the sole carbon source (which supports all 3) at the same initial cell concentration as the co-cultures. As this treatment contained a single, known metabolite, it served as a control for co-culture transcriptome analysis.

Bacterial cell numbers were determined by flow cytometry. Samples were fixed at a final concentration of 1% glutaraldehyde, incubated at 4 °C for 20 min, and stored at −80 °C. Just prior to analysis, an internal standard of 5-μm fluorescent particles (ACFP-50-5; Spherotech, Lake Forest, IL, USA) was added, followed by staining for 15 min with SYBR Green I (final concentration 0.75X; Life Technologies, Waltham, MA, USA). Samples were analyzed on an Agilent Quanteon flow cytometer (Acea, Biosciences Inc, San Diego CA) with a 405 nm laser using a 530/30 bandpass filter for SYBR Green (bacteria) and a 695/40 bandpass filter for chlorophyll *a* (phytoplankton).

### RNAseq analysis

RNA was extracted as in Ferrer-González et al. [33]. Briefly, filters were cut into pieces under sterile conditions and incubated at room temperature for 1 h in TE buffer, SDS (0.6% final concentration), and proteinase K (120 ng μl ^–1^ final concentration), and extracted in equal volumes of acid phenol:chloroform:isoamyl alcohol (25:24:1) and then chloroform:isoamyl alcohol (24:1). Following centrifugation, the resulting supernatant was mixed with 1 volume of isopropanol and sheared by passage through a 21 G syringe needle. Samples were incubated overnight at −20 °C and then centrifuged. The pellet was resuspended in RNase-free water. Potential traces of DNA were removed using the Turbo DNA-free kit (Invitrogen, Waltham, MA, USA), and samples were tested for residual DNA by a 35-cycle PCR targeting the 16S rRNA gene of the strains (27F, 1492R primer set, temperature program: 30 s at 98 °C, 35 cycles of 30 s at 95 °C, 30 s at 50 °C, and 60 s at 72 °C, followed by 15 min at 72 °C). The DNA was processed for sequencing with the NEBNext rRNA Depletion Kit (E7860; New England Bio Labs, Ipswich, MA), modified to remove *Micromonas* and bacterial rRNA using a custom pool of 160 oligonucleotide probes targeting the 18S, 5.8S, and 28S rRNA genes and ITS regions (developed using the NEBNext Custom RNA Depletion Design Tool; https://depletion-design.neb.com) (Table S1) synthesized by Integrated DNA Technologies (Coralville, Iowa, USA) and incorporated into the depletion kit protocol according to manufacturer’s recommendations. Library preparation was carried out using the NEBNext Ultra II Directional Kit (E7765), and libraries were sequenced at the Georgia Genomics and Bioinformatics Core (Athens, GA, USA) on the NextSeq 2000 platform (SE100; Illumina, San Diego, CA, USA). Raw data were deposited in the NCBI SRA BioProject database under accession PRJNA787291, and data product files are available on Zenodo (10.5281/zenodo.6812122).

Quality control of sequences was performed using the FASTX toolkit, imposing a minimum quality score of 20 over 80% of read length. Reads aligning to an in-house rRNA database (10.5281/zenodo.6812122) were removed (SortMeRNA 2.1-GCCcore-8.3.0). Remaining reads were mapped to the *R. pomeroyi* DSS-3, *Stenotrophomonas* sp. SKA14, or *P. dokdonensis* MED152 genome (Bowtie 2) and counted (HTSeq) [34, 35] (NCBI RefSeq accession numbers ASM1196v2, ASM15857v1, and ASM15294v2, respectively), with 51% mapping. Genes with differential expression compared to the glucose controls were determined using DESeq2 [36], with significance after adjusting for multiple comparisons (padj) set at ≤0.01, with *n* = 2, 3, or 4. Annotations of the bacterial genomes were updated by comparison to closely related genomes in JGI Integrated Microbial Genomes and Microbiomes (IMG/M) [37], NCBI RefSeq [38], and Price et al. [39]. The dbCAN web resource was used for identification of carbohydrate-active enzyme annotations [40], considering results of HMMs, peptide pattern recognitions, and protein alignments (Tables S2–S4). For the *M. commoda* RCC299 genome, the gene model sets and functional annotations were obtained from the most recent assembly [41] through the JGI PhycoCosm genome browser [42], referenced against Worden et al. [43] and Guo et al. [44], and verified by BLAST alignments utilizing the JGI IMG database [37] (Tables S5–S7).

## Results and discussion

Ecologically relevant metabolites released extracellularly by the picophytoplankter *M. commoda* RCC299 were postulated based on gene expression responses from three individually co-cultured marine heterotrophic bacteria. The *Micromonas* exometabolites accumulating during 7 d of axenic growth prior to inoculation were the sole carbon source for the bacterial strains. The bacteria represent three taxa commonly found associated with natural marine phytoplankton: Rhodobacteraceae (represented by *R. pomeroyi*), Gammaproteobacteria (*Stenotrophomonas* sp.), and Flavobacteriales (*P. dokdonensis*). These same species were used as biological sensors of *Thalassiosira pseudonana* CCMP1335 exometabolites in a previous study [33]. Bacterial gene expression was determined by sampling 8 h after inoculation and comparing expression when co-growing with the phytoplankter to that on a single metabolite control (400 μM C as glucose). Full information on gene expression is given in Tables S2–S7, but here we focus analysis on transporters and initial steps in catabolic pathways [45, 46], indicative of bacterial detection and utilization of organic compounds in the exometabolite pool, and on carbohydrate-active enzymes (CAZymes) [47], indicative of sugar and glycoconjugate catabolism. Thus, we interpreted bacterial gene expression changes as biological sensors that predict the composition of exometabolites being released from living *Micromonas* cells.

### *Ruegeria pomeroyi* DSS-3 transcriptional responses

**Fig. 1:**
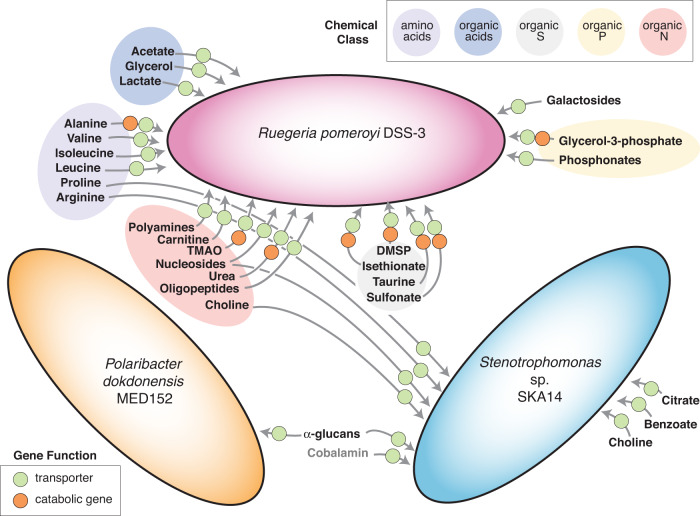
Metabolites hypothesized to mediate autotroph-heterotroph carbon transfer for three model marine bacteria in co-culture with *M. commoda*. Circles specify genes which enable transport (green) or catabolism (orange). Shaded ovals identify chemical classes of metabolites. Cobalamin (vitamin B_12_) was added to the growth medium (light gray font).

We found 513 of the 4332 *R. pomeroyi* protein-encoding genes significantly enriched in the *Micromonas* co-culture relative to the glucose control (2- to 216-fold; DeSeq2, padj ≤0.01), representing 12% of coding sequences in the bacterium’s genome (Table S2). Expression of transporters, catabolic genes diagnostic of initial degradation of known compounds, and CAZymes accounted for 103 of the significantly enriched *R. pomeroyi* genes (2.4% of all genes; Table 1). Twenty-seven transporter systems annotated for uptake of five chemical classes of organic compounds were among the differentially expressed genes. The classes included essential amino acids (leucine, isoleucine, valine, and alanine); organic sulfur compounds [dimethylsulfoniopropionate (DMSP), isethionate, taurine, and *N*-acetyltaurine]; organic acids (glycerol, lactate, and malate); carbohydrates (glycosides, galactosides; and glycerol-3-phosphate), and a nitrogen-containing osmolyte [trimethylamine N-oxide (TMAO)] (Fig. 1). Other predicted extracellular metabolites available to *R. pomeroyi* included polyamines, oligopeptides, nucleosides, and urea (Table 1). Seven of these compounds were found previously by direct chemical analysis in the endometabolome of close relative *Micromonas pusilla*: leucine, isoleucine, DMSP, malate, glycerol-3-phosphates, nucleosides, and polyamines [23]. Six were recently confirmed experimentally by analysis of *R. pomeroyi* transporter mutants [48] (Table 1).

**Table 1:** Significantly enriched genes for transporter systems, catabolic enzymes, and carbohydrate-active enzymes in the transcriptome of *R. pomeroyi DSS-3* in co-culture with *M. commoda*.

Locus Tag	Protein ID	Fold Change	Description
Transporters			
SPO0237	AAV93562.1	36.3	SN-glycerol-3-phosphate ABC transporter, ATP-binding protein *ugpC*
SPO0238	AAV93563.1	25.4	SN-glycerol-3-phosphate ABC transporter, permease *ugpE*
SPO0239	AAV93564.1	30.3	SN-glycerol-3-phosphate ABC transporter, permease *ugpA*
SPO0240	AAV93565.1	23.7	SN-glycerol-3-phosphate ABC transporter, substrate-binding *ugpB*
SPO0674*	AAV93982.1	2.2	taurine ABC transporter, substrate-binding protein *tauA*
SPO0675	AAV93983.1	3.9	taurine ABC transporter, ATP-binding protein *tauB*
SPO0676*	AAV93984.1	2.2	taurine ABC transporter, permease *tauC*
SPO0703	AAV94009.1	2.6	oligopeptide ABC transporter, ATP-binding protein
SPO0704	AAV94010.1	2.7	oligopeptide ABC transporter, permease
SPO0705	AAV94011.1	2.8	oligopeptide ABC transporter, permease
SPO0706	AAV94012.1	2.8	oligopeptide ABC transporter, substrate-binding protein
SPO0736	AAV94041.1	2	TRAP dicarboxylate transporter, large permease
SPO0780	AAV94085.1	3.6	phosphonate ABC transporter, ATP-binding protein *phnC*
SPO0781	AAV94086.1	2.5	phosphonate ABC transporter, substrate-binding protein *phnD*
SPO0782	AAV94087.1	4.1	phosphonate ABC transporter, permease *phnE*-1
SPO0783	AAV94088.1	2.8	phosphonate ABC transporter, permease *phnE*-2
SPO1548	AAV94835.1	6.5	TMAO transporter, substrate-binding protein *tmoX*
SPO1549	AAV94836.1	6.8	TMAO transporter, ATP-binding protein *tmoW*
SPO1550	AAV94837.1	6.4	TMAO transporter, permease *tmoV*
SPO2356	AAV95617.1	3.7	isethionate TRAP transporter, large permease *iseM*
SPO2357*	AAV95618.1	3.5	isethionate TRAP transporter, small permease *iseL*
SPO2358*	AAV95619.1	9.3	isethionate TRAP transporter, substrate-binding protein *iseK*
SPO2370	AAV95630.1	3	sodium:alanine symporter family protein
SPO2699	AAV95944.1	5.4	opine/polyamine ABC transporter, permease
SPO2700	AAV95945.1	3.2	opine/polyamine ABC transporter, permease
SPO2701	AAV95946.1	3.1	opine/polyamine ABC transporter, substrate binding protein
SPO2702	AAV95947.1	3.9	opine/polyamine ABC transporter, ATP-binding protein
SPO3186*	AAV96421.1	2.5	DMSP transporter
SPO3472	AAV96698.1	2.6	polyamine ABC transporter, ATP-binding protein
SPO3783	AAV97003.1	4.7	sugar ABC transporter, ATP-binding protein
SPO3784	AAV97004.1	4.6	sugar ABC transporter, ATP-binding protein
SPO3785	AAV97005.1	3.9	sugar ABC transporter, permease
SPO3786	AAV97006.1	5.6	sugar ABC transporter, permease
SPO3787	AAV97007.1	5.2	sugar ABC transporter, substrate-binding protein
SPOA0278	AAV97412.1	7.5	TRAP dicarboxylate transporter, large permease *dctM*
SPOA0279	AAV97413.1	7.8	TRAP dicarboxylate transporter, small permease *dctQ*
SPOA0280	AAV97414.1	13.3	TRAP dicarboxylate transporter, substrate-binding protein *dctP*
SPOA0296	AAV97430.1	1.9	branched-chain amino acid ABC transporter, ATP-binding *livF*-2
SPOA0297	AAV97431.1	2.1	branched-chain amino acid ABC transporter, ATP-binding *livG*
SPOA0299	AAV97433.1	2	branched-chain amino acid ABC transporter, permease
SPOA0300	AAV97434.1	2.1	branched-chain amino acid ABC transporter, substrate binding
SPOA0374	AAV97506.1	2.2	TRAP dicarboxylate transporter, substrate-binding protein *dctP*
SPOA0381	AAV97513.1	2.4	spermidine/putrescine ABC transporter, substrate-binding protein
SPOA0384	AAV97516.1	2.8	spermidine/putrescine ABC transporter, permease
SPO1848	AAV95127.1	2.9	branched-chain amino acid ABC transporter, ATP binding protein
SPO1849	AAV95128.1	2	branched-chain amino acid ABC transporter, ATP binding protein
SPO1850	AAV95129.1	2	branched-chain amino acid ABC transporter, permease
SPO2626*	AAV95871.1	3.8	fumarate, succinate, malate TRAP transporter, *dctM*
SPO2627	AAV95872.1	3.2	fumarate, succinate, malate TRAP transporter, *dctQ*
SPO2628*	AAV95873.1	2.3	fumarate, succinate, malate TRAP transporter, *dctP*
SPO0608*	AAV93923.1	7.1	glycerol ABC transporter, substrate binding protein
SPO0609	AAV93924.1	12.2	glycerol ABC transporter, ATP binding protein
SPO0610	AAV93925.1	8.5	glycerol ABC transporter, ATP binding protein
SPO0611	AAV93926.1	12.6	glycerol ABC transporter, permease
SPO0612	AAV93927.1	10.5	glycerol ABC transporter, permease
SPO1017	AAV94321.1	5.7	lactic acid ABC transporter ATP-binding protein
SPO1018	AAV94322.1	6.6	lactic acid ABC transporter ATP-binding protein
SPO1019	AAV94323.1	4.5	lactic acid ABC transporter, permease
SPO1020	AAV94324.1	3.6	lactic acid ABC transporter, permease
SPO1021	AAV94325.1	3.7	lactic acid ABC transporter, substrate binding protein
SPO1485	AAV94772.1	2.4	sodium:galactoside symporter family protein
SPO1707	AAV94990.1	3.4	urea ABC transporter, ATP binding protein
SPO1707a	AAV97140.1	4.6	urea ABC transporter, ATP binding protein
SPO1708	AAV94991.1	5.8	urea ATP transporter, permease
SPO1709	AAV94992.1	7.9	urea ATP transporter, permease
SPO1710	AAV94993.1	6.4	urea ABC transporter, substrate binding protein
SPO1810	AAV95089.1	2.9	sodium/solute symporter family protein (acetate, putative)
SPO1848	AAV95127.1	2.9	branched-chain amino acid ABC transporter, ATP-binding protein
SPO1849	AAV95128.1	2	branched-chain amino acid ABC transporter, ATP-binding protein
SPO1850	AAV95129.1	2	branched-chain amino acid ABC transporter, permease
SPO2658	AAV95903.1	2.3	possible sulfonate ABC transporter, substrate binding protein
SPO2660	AAV95905.1	2.6	possible sulfonate ABC transporter, permease
SPO2661	AAV95906.1	2.1	possible sulfonate ABC transporter, ATP binding protein
SPO2802	AAV96043.1	2.3	nucleoside ABC transporter, substrate binding protein
SPO2803	AAV96044.1	4.1	nucleoside ABC transporter, ATP binding protein
SPO2804	AAV96045.1	3.1	nucleoside ABC transporter, permease
SPO2805	AAV96046.1	3.1	nucleoside ABC transporter, permease
SPO3290	AAV96517.1	2.2	branched-chain amino acid ABC transporter, ATP-binding protein
CAZymes			
SPO1205	AAV94501.1	3.1	UDP-3-0-acyl N-acetylglucosamine deacetylase (CE11)
SPO3555	AAV96780.1	2.1	transglycosylase (GH23)
SPO2587	AAV95835.1	2.4	penicillin-binding protein 1 A (GT51)
SPO0898	AAV94203.1	2.4	hypothetical protein (GT0)
SPO3258	AAV96493.1	2.9	glycosyl hydrolase (GH25)
SPO0190	AAV93516.1	2.1	choline oxidoreductase (AA3)
Organic Sulfur Utilization			
SPO1913	AAV95190.1	2	dimethylsulfoniopropionate demethylase (*dmdA*)
SPO2045	AAV95316.1	1.9	3-methylmercaptopropionyl-CoA ligase (*dmdB*2)
SPO3804	AAV97018.1	12.4	3-methylmercaptopropionyl-CoA dehydrogenase (*dmdC*)
SPO3805	AAV97019.1	11.3	methylthioacryloyl-CoA hydratase (*dmdD*)
SPOA0318	AAV97450.1	28.9	methionine gamma-lyase (*megL*)
SPO0673	AAV93981.1	2.7	taurine-pyruvate aminotransferase (*tpa*)
SPO0658	AAV93966.1	2.9	*N*-acetyltaurine amidohydrolase (naaS)
SPO2359	AAV95620.1	10.3	isethionate dehydrogenase (*iseJ*)
SPO3560	AAV96785.1	19.3	phosphate acetyltransferase (*pta*)
SPO3561	AAV96786.1	27.2	sulfoacetaldehyde acetyltransferase (*xsc*)
SPO2657	AAV95902.1	3.1	sulfolyase (*cuyA2*)
Organic Nitrogen Utilization			
SPO1562	AAV94849.2	1.5	trimethylamine-oxide aldolase (*tdm*)
SPO1579	AAV94866.1	6.3	dimethylamine monooxygenases submit (*dmmD*)
SPO1580	AAV94867.1	5.5	dimethylamine monooxygenases submit (*dmmA*)
SPO1581	AAV94868.1	5.8	dimethylamine monooxygenase subunit (*dmmB*)
SPO1582	AAV94869.1	4.9	dimethylamine monooxygenase subunit (*dmmC*)
SPO0222	AAV93547.1	14.6	alanine dehydrogenase (*ald*)
SPO1711	AAV94994.1	2.9	urease accessory protein (*ureD*)
SPO1712	AAV94995.1	3.3	urease gamma subunit (*ureA*)
SPO1713	AAV97135.1	3.1	urease beta subunit (*ureB*)
Organic Phosphate Utilization			
SPO0732	AAV94037.1	6.3	aerobic glycerol-3-phosphate dehydrogenase (*glpD*)

All genes have an adjusted *p*-value ≤ 0.01.

*GH* glycoside hydrolase, *AA* auxiliary activity, *GT* glycosyl transferase, *CE* carbohydrate esterase.

*Function confirmed with mutant strain.

In some cases, enriched genes in diagnostic catabolic pathways supported the signals from transporters; they included genes for the catabolism of TMAO (*dmmDABC*), alanine (*ald*), DMSP (*dmdACD*, *megL*), taurine (*tpa, xsc, pta*), isethionate (*iseJ*), glycerol-3-phosphate (*glpD*) and urea (*ureABD*), many of which have been shown to be assimilated by *R. pomeroyi* [49–53]. In one case, the catabolic gene *N*-acetyltaurine amidohydrolase (*naaS)*, specific for the catabolism of *N*-acetyltaurine, added a new compound to the suite of exometabolites hypothesized from transporter expression (Table 1). CAZyme genes enriched in the transcriptome indicated catabolism or modifications of peptidoglycan and its components (CAZyme annotations CE11, GH23, GH25, GT51; Table 1). The full biosynthesis pathway for peptidoglycan synthesis is present in the closely-related species *M. pusilla* CCMP1545 (although peptidoglycan itself has not been detected), while the *M. commoda* pathway appears incomplete [54]. Nonetheless, several genes present in the *M. commoda* genome could generate peptidoglycan intermediates (MURE, MRAY, MURG, and PBP). Finally, one *R. pomeroyi* CAZyme with higher relative expression was annotated for the oxidation of the osmolyte choline (AA3), which *R. pomeroyi* can use as a substrate [55].

In the glucose control, 219 *R. pomeroyi* genes were significantly enriched (2- to 75-fold; DeSeq2, padj ≤0.01, Table S2). As expected, enriched genes included those encoding glucose transport and metabolism to pyruvate via glycolysis and the Entner-Doudoroff pathway.

### *Stenotrophomonas* sp. SKA14 transcriptional responses

We found 337 of the 4469 *Stenotrophomonas* sp. protein-encoding genes with significantly higher relative expression in the *Micromonas* culture relative to the glucose control (2- to 33-fold; DeSeq2, padj ≤0.01), representing 7.5% of coding sequences in the bacterium’s genome (Table S3). Expression of transporters, diagnostic catabolic genes, and CAZymes accounted for 20 of the significantly enriched genes (0.5% of all genes; Table 2). The *Stenotrophomonas* sp. genome contains 9 enriched organic compound transporters that do not yet have a definitive functional assignment. One is an enriched TonB-dependent transporter (TBDT), a component of a polysaccharide utilization locus-like (PUL-like) structure with a putative annotation for the degradation of α-glucans (Table 2). Others included a transporter that potentially mediates the uptake of nucleosides (*nupC*), and a transporter with a putative annotation for uptake of nucleobases xanthine and uracil. Putative osmolyte transporters with differential expression included a betaine-choline-carnitine transporter (BCCT) gene predicted for choline uptake (*betT*), and a major facilitator superfamily (MFS) transporter predicted for proline uptake (*proP*). Finally, two TBDT transporters with higher relative expression are annotated as potential cobalamin (vitamin B_12_) receptors. The *Stenotrophomonas* sp. genome does not contain genes for de novo B_12_ biosynthesis, and therefore the signal from these transporters may represent uptake from the culture medium. Like *R. pomeroyi*, enriched CAZymes in the *Stenotrophomonas* sp. transcriptome are indicative of modification or catabolism of peptidoglycan-like substrates (CBM50 + GH23, GH103, GH103, GH23, CBM50, GT2). Phenotypic studies of *Stentrophomonas* sp. SKA14 are not available, but other members of the genus, including beneficial plant-associated strains [56], share the ability to grow on peptidoglycan monomer *N*-acetylglucosamine and glucose [57].

**Table 2:** Significantly enriched genes for transporter systems, catabolic enzymes, and carbohydrate-active enzymes in the transcriptome of *Stenotrophomonas* sp.

Locus Tag	Protein ID	Fold Change	Description
Transporters			
SSKA14_135	WP_008264570.1	4.3	MFS transporter
SSKA14_1119	WP_008265489.1	2.4	TonB-dependent receptor (α-glucan PUL)
SSKA14_516	WP_006450256.1	2.4	high-affinity choline transporter, *betT*
SSKA14_2109	WP_008266462.1	2.2	proline/glycine betaine transporter, *proP*
SSKA14_993	WP_040009025.1	3.3	TonB-dependent receptor, cobalamin
SSKA14_3190	WP_040007328.1	2.6	TonB-dependent receptor, cobalamin
SSKA14_2274	WP_004154263.1	2.5	nucleoside transporter, *nupC*
SSKA14_4041	WP_008268362.1	2.3	NCS2 family permease: Xanthine/Uracil Family Permease
SSKA14_1406	WP_006461445.1	2.2	arginine/agmatine antiporter
CAZymes			
SSKA14_2639	WP_008267014.1	2.7	lytic transglycosylase (CBM50 + GH23)
SSKA14_RS05295	WP_040007079.1	2.2	lytic murein transglycosylase B (GH103)
SSKA14_3712	WP_008268057.1	2.1	lytic murein transglycosylase (GH23)
SSKA14_1720	WP_008266069.1	1.9	LysM peptidoglycan-binding domain-containing protein (CBM50)
SSKA14_2822	WP_040007055.1	5.3	glucans biosynthesis glucosyltransferase MdoH (GT2)
SSKA14_433	WP_040009049.1	3.3	lytic transglycosylase domain-containing protein (GH23)
α-glucan PUL			
SSKA14_1119	WP_008265489.1	2.4	TonB-dependent receptor

SKA14 in co-culture with *M. commoda*. All genes have an adjusted *p* value ≤ 0.01.

*GH* glycoside hydrolase, activity, *GT* glycosyl transferase, *CBM* carbohydrate binding module.

In the glucose control, 358 genes were significantly enriched (2- to 19-fold; DeSeq2, padj ≤0.01; Table S3) compared to *Stenotrophomonas* in co-culture. Glucose transporter genes are not annotated in the *Stenotrophomonas* sp. genome, and catabolism of glucose through glycolysis and the pentose phosphate pathway did not stand out in the transcriptional data. We suspect that uptake and metabolism of α-1,4-glucan monomers released by *Micromonas* led to similar expression levels of the glucose transporter and glycolysis genes in both the co-culture and control transcriptomes.

### *Polaribacter dokdonensis* MED152 transcriptional responses

We found 185 of the 2614 *P. dokdonensis* protein-encoding genes significantly enriched in the *Micromonas* co-culture relative to the glucose control (2- to 38-fold; DeSeq2, padj ≤0.01), representing 7% of coding sequences in the bacterium’s genome (Table S4). Expression of transporters, diagnostic catabolic genes, and CAZymes accounted for 11 of the significantly enriched genes (0.4% of all genes; Table 3). There were no transporter systems with annotations for uptake of organic compounds among the genes with significantly higher relative expression. There were five CAZymes enriched, however, including two in PUL3 (as designated in the CAZy database): a trehalase (GH37) for hydrolysis of the α-glucosidic linkages of α,α-trehalose, and an amylo-1,4 → 1,6-transglucosidase (GH13_18) that transfers a segment of a α-1,4-glucan chain. These two enzymes are suggestive of the presence of a α-1,4-glucan in the exometabolome of *Micromonas*. Members of the green algal lineage, like their true plant relatives, carry genes for carbon storage as starch, consisting of α-1,4-glucan units as amylose and amylopectin [58, 59]. In PUL5, enriched genes included an *N*-acetylglucosamine kinase. Two other CAZymes in this PUL, β-*N*-acetylhexosaminidase (GH20) and glucosamine-6-phosphate deaminase, had annotations for *N*-acetyl-D-glucosamine catabolism, although these were significant at a less stringent cutoff of padj ≤0.05 (Table 3). These results suggest that *P. dokdonensis* had access to *N*-acetylglucosamine, consistent with evidence from the other two bacteria that components of a peptidoglycan-like polymer were available in the *Micromonas* exometabolome. Other enriched CAZymes not located within PULs included a glycosyl transferase (GT2) and an endo-β-*N*-acetylglucosaminidase (GH163). The transcriptional patterns found here are consistent with previous findings of very low numbers of amino acid and sugar transporters in the *P. dokdonensis* genome, suggesting specialization for growth instead on polymeric substrates (polysaccharides and proteins) [60].

**Table 3:** Significantly enriched transporter system genes and carbohydrate-active enzymes in the transcriptome of *Polaribacter dokdonensis* MED152 in co-culture with *M. commoda* RCC299.

Locus Tag	Protein ID	Fold Change	Description
CAZymes			
MED152_03905	WP_015480551.1	8.8	glycosyltransferase family 2 protein (GT2)
MED152_05075	WP_015480781.1	7.4	amylo-(1,4 to 1,6) transglucosidase (GH13_8)
MED152_05110	WP_015480788.1	4.7	trehalase (GH37)
MED152_03915	WP_015480553.1	18.5	DUF4838 domain-containing protein (GH163)
MED152_03105	WP_041383822.1	6	response regulator (GT2)
PUL3			
MED152_05075	WP_015480781.1	7.4	amylo-(1,4 to 1,6) transglucosidase (GH13_8)
MED152_05080	WP_041383396.1	5.7	sugar kinase
MED152_05110	WP_015480788.1	4.7	trehalase (GH37)
PUL5			
MED152_08485	WP_015481446.1	2.6	glucosamine-6-phosphate deaminase
MED152_08490	WP_015481447.1	3.2	beta-N-acetylhexosaminidase (GH20, PUL5)
MED152_08495	WP_015481448.1	4.5	N-acetylglucosamine kinase

All genes have an adjusted *p* value ≤ 0.01.

*GH* glycoside hydrolase, *AA* auxiliary activity, *CE* carbohydrate esterase, *GT* glycosyl transferases.

In the *P. dokdonensis* glucose control, 236 genes were significantly enriched (2- to 30-fold; DeSeq2, padj ≤0.01). These included the genes that encode glucose transport and metabolism via glycolysis (Table S4).

### *Micromonas commoda* RCC299 transcriptional responses

**Fig. 2:**
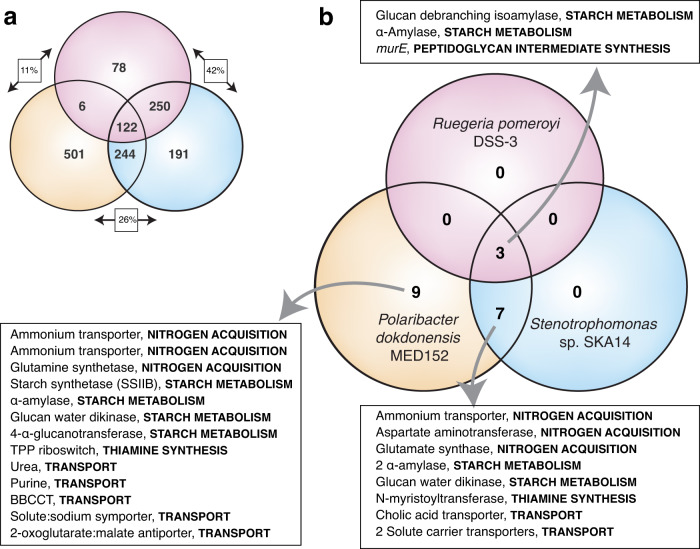
*M. commoda* transcript enrichment in the presence of one or more of the three heterotrophic bacteria. **a** Transcription was enriched for 1393 genes in *Micromonas* across all treatments. Boxed values indicate pair-wise comparisons of the genes enriched in both species as a percent of the genes enriched in only one or the other. Complete gene information and fold-change values are given in Tables S5, S6, and S7. Significance was determined based on padj values ≤0.01. **b** Transcription was enriched for 19 potential substrate-related genes in *Micromonas*, 10 of which were found for more than one co-cultured bacterium. Complete gene information and fold-change values are given in Table S8.

Of the 10,240 *Micromonas* protein-encoding genes, 456 were significantly enriched in the presence of *R. pomeroyi* relative to the axenic control (DeSeq2, padj ≤0.01), with 78 unique to this co-culture. In the *Stenotrophomonas* sp. co-culture, 807 genes were significantly enriched, with 191 unique. In the *P. dokdonensis* co-culture, 873 genes were significantly enriched, with 501 unique (Fig. 2a; Tables S5, 6, 7). We identified five categories of enriched genes with potential relevance to the metabolite interactions inferred from the bacterial transcription: starch metabolism, synthesis of peptidoglycan-like intermediates, organic compound transport, thiamine synthesis, and nitrogen acquisition (Fig. 2b; Table S8). Seven enriched *Micromonas* genes function in starch metabolism. Two of these responded to the presence of all three bacteria: the debranching enzyme isoamylase (ISA1) that removes wrongly positioned branches during starch biosynthesis, and the α-amylase AMYA2 that metabolizes branched starch into linear glucans during breakdown [58] (Fig. 2). Other enriched starch-related enzymes were the glucan water dikinase (GWD2) which phosphorylates and catalyzes degradation of starch [58, 61, 62], two other α-amylases (AMYA3 and 6), a starch synthase (SSIIB) [58], and a 4-α-glucanotransferase (DPE) that breaks down linear glucans to maltose [58] (Table S8). These changes in relative gene expression suggest that *Micromonas* activated starch metabolism components such as amylopectin, and they complement the enriched bacterial catabolic genes for α-1,4-glucan degradation.

In all three bacterial co-cultures compared to the axenic culture, relative increases in *Micromonas* expression was observed for UDP-*N*-acetylmuramoylalanyl-D-glutamate-2,6-diaminopimelate ligase (*murE*), an enzyme in the green alga’s incomplete peptidoglycan-like biosynthesis pathway (Fig. 2b; Table S8). This is consistent with the enriched signals of peptidoglycan catabolism or modification in all three bacterial transcriptomes, but whether the substrates inducing the bacterial enzymes came from bacterial or phytoplankton metabolite pools could not be distinguished. A number of enriched *Micromonas* transporter proteins were annotated for targeting organic substrates. Their direction of transport is not known, however, and they could either import or export compounds. Predicted substrates of the transporters are purines, betaine/carnitine/choline, cholic acid, and urea (Table S8). All have been reported previously as components of marine phytoplankton endometabolomes [63–65], and all except cholic acid complement transcription signals by one or more of the co-cultured bacteria.

A subset of the *Micromonas* transporter genes enriched in the presence of bacteria were annotated for mediating the uptake or assimilation of exogenous compounds, potentially of bacterial origin (Table S8). A gene that encodes a TPP riboswitch regulating thiamine biosynthesis (UNK1) was enriched in the presence of *Stenotrophomonas* sp. and *P. dokdonensis*. Previous studies in *M. commoda* have shown this gene to be activated in response to thiamine depletion [66], and could indicate depletion in the co-culture. Correspondingly, an *N*-myristoyltransferase 1 (NMT1) [43] associated with thiamine biosynthesis was enriched in the presence of *P. dokdonensis*. Another key transcriptional signal consisted of 6 enriched genes related to nitrogen acquisition. The co-culture medium contained nitrate as the only nitrogen source, which *Micromonas* can use. Nonetheless, in the presence of two bacterial species (*Stenotrophomonas* sp. and *P. dokdonensis*) the picoeukaryote’s transcriptome was enriched for ammonium transporters, glutamate synthase GSN1 and glutamine synthetase GSIII (key components of the glutamine synthetase–glutamate synthase cycle or GS-GOGAT system for ammonium incorporation) [67, 68], and the aspartate aminotransferase ASP1 (which transaminates glutamate and oxaloacetate to 2-oxoglutarate and aspartate reversibly, depending on nitrogen availability) [67, 69]. The activation by *M. commoda* of these uptake and metabolism genes suggests that the bacteria induced a shift in nitrogen acquisition strategies that did not occur under axenic conditions.

### Bacterial sensing of *Micromonas* versus *Thalassiosira* exometabolites

A previous study of metabolite availability with these same three bacteria in co-culture with *Thalassiosira pseudonana* CCMP1335 [33] allows for a comparison of the biologically-sensed substrates between a representative picoeukaryote and diatom (Tables S9, S10, S11). Transporters or catabolic genes that were induced in one or more bacterial species when growing on the exometabolome of each phytoplankter indicated overlap in composition for the metabolites glycerol, sn-glycerol-3-phosphate, lactate, nucleosides, urea, and proline. Three organic sulfur compounds were also predicted to be shared between the phytoplankton exometabolomes; these are DMSP, *N*-acetyltaurine, and an unidentified sulfonate.

Bacterial transcription signals also suggested that several metabolites were unique to the *Micromonas* metabolome. These included the organic sulfur compound isethionate, organic nitrogen compounds TMAO, choline, carnitine, and polyamines, and organic sulfur and nitrogen compound taurine. Expression signals unique to the *Thalassiosira* exometabolome were the organic sulfur compound 2,3-dihydroxypropane-1-sulfonate (DHPS) and the organic nitrogen compounds glutamine, *N*-acetyl-galactosamine, and peptides. Other unique *Thalassiosira* compounds suggested by bacterial expression were benzoate, citrate, β-1,3-glucans, and an alginate-like oligosaccharide.

**Fig. 3:**
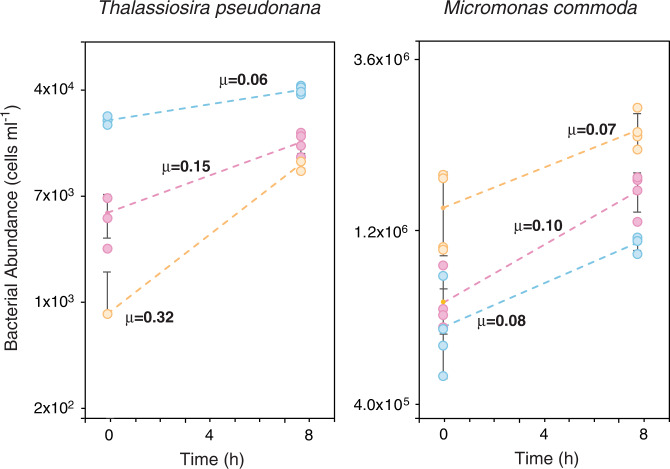
Bacterial growth during 8 h incubations with microphytoplankter *Thalassiosira pseudonana* and picoeukaryotic phytoplankter *Micromonas commoda*. Replicates (*n* = 4) are plotted individually, and standard errors of the means are indicated. Specific growth rates (μ, in units of h^−1^) are estimated from the T0 and T8 cell numbers, assuming exponential growth. Pink symbols, *Ruegeria pomeroyi* DSS-3; blue symbols, *Stenotrophomonas* sp. SKA14; orange symbols, *Polaribacter dokdonensis* MED152. Initial phytoplankton exometabolite pools consisted of organic compounds released during 7 d of axenic culture (exponential through early stationary phase).

A comparison of bacterial cell numbers at the beginning and end of the experiment indicated that all three bacteria increased in abundance during the 8 h co-cultures with phytoplankton (T-test; *p* < 0.01) (Fig. 3). *R. pomeroyi* grew at a faster rate than the other bacteria in *Micromonas* exometabolites (μ = 0.10 h^−1^), while *P. dokdonensis* grew faster rate than the others in *Thalassiosira* exometabolites (μ = 0.32 h^−1^), emphasizing that exometabolite composition differentially affected bacterial species’ fitness relative to one another.

## Conclusions

Identifying the specific compounds responsible for microbial carbon flux in surface ocean environments is challenging from a chemical analysis perspective because of their low concentrations, rapid uptake, and partitioning with sea salts in extraction protocols [70]. Here we used transcriptional signals of bacteria representative of phytoplankton-associated taxa to postulate molecules mediating microbial carbon flux. This approach assumes that the expression of bacterial transport or catabolism genes indicate the presence of their target metabolite, which is generally the case [33, 71]. Increased transporter expression in the absence of substrates can be energetically unfavorable for bacteria [72], and marine bacterial communities have been shown experimentally to respond to the availability of new substrates by increasing transporter expression [73–75]. However, caveats to this approach include posttranscriptional or constitutive gene regulation, and incorrect bacterial gene annotation (Table S1, S2, and S3) [76].

The *Micromonas* exometabolome is hypothesized to include at least 38 metabolites based on transcriptional regulation by the bacteria, many of which contained the heteroatoms nitrogen (55%), sulfur (13%), or phosphorus (8%). There was evidence of resource partitioning among the three heterotrophic bacteria, as was also found earlier for the *Thalassiosira* exometabolome [33]. For example, enrichment of transcripts for uptake of low molecular weight organic sulfur compounds was unique to *R. pomeroyi*, agreeing with previous findings that utilization of phytoplankton-derived sulfur metabolites is characteristic of marine Rhodobacteraceae [6, 51, 77]. In contrast, both *Stenotrophomonas* and *P. dokdonensis* had higher proportions of significant genes annotated for catabolism of α-glucans compared to *R. pomeroyi*.

Comparative analysis of hypothesized metabolite release by *M. commoda* and *T. pseudonana* based on gene expression patterns of the co-cultured bacteria indicated that *Micromonas* exometabolites overlapped with the diatom’s at the level of broad chemical categories (such as fatty acids, nucleosides, amino acids, and organic sulfur compounds), but differed in the specific compounds within those categories. For example, bacterial gene expression predicts that *Micromonas* is a more important source of the organic sulfur compounds taurine and isethionate and of the methylamines TMAO and choline, but a less important source of DHPS, glutamine, and oligosaccharides. Several of these metabolites are hypothesized to be used selectively by marine bacterial taxa; for instance, DHPS catabolism genes are concentrated in specific groups of marine Alphaproteobacteria that also have complete biosynthesis pathways for B_12_ [71], a vitamin which is required yet not synthesized by *M. commonda* based genome analysis and experimental data from close relative *M. pusilla* [78]. On the other hand, degradation pathways for diverse oligosaccharides are common in Flavobacteriia and Gammaproteobacteria [79, 80]. Continued increases in surface ocean temperature coupled to decreases in nutrient supply [16, 25] are predicted to favor smaller phytoplankton (e.g., picoeukaryotes such as *Micromonas*) over larger phytoplankton (i.e., microphytoplankton such as diatoms) [21, 30]. Our results suggest that phytoplankton community shifts will cascade to changes in the pool of microbial-derived metabolites, potentially selecting for different communities of associated heterotrophic bacteria. Given that the movement of carbon from phytoplankton to bacteria via the labile DOC pool is one of the largest annual organic carbon transfers in the ocean [70], future modifications to this flux may have important consequences to global biogeochemical cycles.

## Supplementary information

Supplementary Tables

## Data Availability

Raw data are deposited in the NCBI SRA BioProject database under accession PRJNA787291, and data product files are available on Zenodo (10.5281/zenodo.6812122).
